# Relationship between glycemic control and histochemical myeloperoxidase activity in neutrophils in patients with type 2 diabetes

**DOI:** 10.1186/s13098-015-0115-3

**Published:** 2015-12-30

**Authors:** Mustafa Unubol, Irfan Yavasoglu, Firuzan Kacar, Engin Guney, Imran Kurt Omurlu, Mevlut Ture, Gurhan Kadikoylu, Zahit Bolaman

**Affiliations:** Department of Endocrinology, Faculty of Medicine, Adnan Menderes University, 09100 Aydın, Turkey; Department of Hematology, Faculty of Medicine, Adnan Menderes University, Aydın, Turkey; Department of Pathology, Faculty of Medicine, Adnan Menderes University, Aydın, Turkey; Department of Biostatistics, Faculty of Medicine, Adnan Menderes University, Aydın, Turkey

**Keywords:** Diabetes mellitus, Myeloperoxidase activity, Glycemic control, Diabetic complications

## Abstract

**Background:**

Myeloperoxidase (MPO) is a lysosomal hemoprotein found in the azurophilic granules in neutrophils. Myeloperoxidase plays an important role in oxygen-dependent killing of bacteria, fungi, virus and malignant cells. Diabetes mellitus (DM) is listed among conditions that may lead to secondary MPO deficiency in neutrophils but inconsistent results concerning MPO activity in diabetic patients have been reported in the literature. In this study, we aimed to evaluate the relationship between glycemic control in patients with type 2 DM and MPO activity in neutrophils from a histochemical perspective.

**Methods:**

The study included 40 patients with type 2 DM with poor glycemic control, 30 patients with type 2 DM with good glycemic control and 31 healthy controls. Peripheral blood smears were analyzed for each patient included in the study. Myeloperoxidase dye was used for staining. Myeloperoxidase ratios in neutrophil were evaluated for proportions of staining with MPO in 100 neutrophils in each smear. SPSS 16.0 version was used for statistical analyses.

**Results:**

Myeloperoxidase ratios in neutrophils were 70 (58.5–80) in type 2 DM patients with poor glycemic control compared to 80 (73.75–90) in those with good glycemic control and 88 (78–92) in healthy controls. The DM group with poor glycemic control was statistically significantly different from the other groups (p < 0.001).

**Conclusions:**

Poor glycemic control in diabetic patients results in decreased MPO activity in neutrophils histochemically.

## Background

Several microbicidal dysfunctions of the neutrophils and monocytes have been defined as a factor that contributes to complications and to morbidity and mortality in diabetic patients [1, 2]. Deficiencies in a number of functions have been demonstrated in the neutrophils of diabetic patients [3–5]. Oxidative stress and reactive oxygen species are known to have important roles in the etiology of diabetes, in development of its complications and in disease progression [6]. Myeloperoxidase (MPO) is a lysosomal hemoprotein found in the azurophilic granules in neutrophils [7]. It is a potent bactericidal enzyme that produces reactive oxygen species. Myeloperoxidase plays an important role in oxygen-dependent killing of bacteria, fungi, virus and malignant cells. Myeloperoxidase-mediated damage is not limited to intraphagosomal microbes. It is involved in the pathogenesis of several inflammatory conditions, atherosclerosis, demyelinating diseases of the central nervous system and some tumors [8–10].

Diabetes mellitus (DM) is listed among conditions that may lead to secondary MPO deficiency in neutrophils [9] but inconsistent results concerning MPO activity in diabetic patients have been reported in the literature [11–13]. Our literature scan demonstrated that the effect of glycemic control in diabetic patients on MPO activity in neutrophils has not been investigated from a histochemical perspective previously.

In this study, we aimed to evaluate the relationship between glycemic control in patients with type 2 DM and MPO activity in neutrophils from a histochemical perspective.

## Methods

### Study design and protocol

This study was designed as an observational, cross-sectional and case–controlled study. Patients with type 2 DM who presented to outpatient clinics of the Adnan Menderes University, Medical Faculty, Department of Endocrinology and Metabolic Disease between January and March 2012 were studied. The patients with type 2 DM applying to the follow-up clinic, and lacking the exclusion criteria, agreeing to participate in the study were enrolled consecutively from January 2012. The study included 40 patients with type 2 DM with poor glycemic control (HbA1c > 7), 30 patients with type 2 DM with good glycemic control (HbA1c ≤ 7) [14] and 31 healthy controls. Patients with active infection, known malignancy, hematologic malignancy, iron deficiency, thrombotic conditions, renal transplantation, patients currently treated with cytotoxic agents, fenofibrate, and anti-inflammatory drugs, and pregnant women were excluded from the study.

**Fig. 1:**
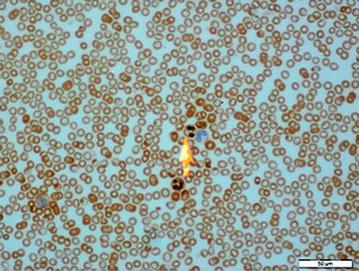
Microscopic image of the neutrophil with positive MPO staining in peripheral blood

Three peripheral blood smears (in fasting period) were analyzed for each patient included in the study. Myeloperoxidase dye was used for staining. Myeloperoxidase ratios in neutrophils (Fig. 1) were evaluated by one hematologist and one hematopathologist for proportions of staining with MPO in 100 neutrophils in each smear, and the mean value of the readings were reported [15].

### Statistical analysis

SPSS 16.0 version was used for statistical analyses. Fit of the quantitative data to normal distribution was studied using the Shapiro–Wilk test. For variables that were fit for normal distribution, one-way variance analysis (ANOVA) was used for statistical comparisons and descriptive statistics were expressed as mean ± standard deviation. For variables unfit for normal distribution, Kruskal–Wallis was used for statistical comparisons and descriptive statistics were expressed as median (25–75 ‰). The level of relationship between variables was studied with Spearman’s Rho correlation coefficient. Chi square test and Fisher test were used in analyzing categorical variables and descriptive statistics were expressed as frequency (%). In our study, we used classification and regression tree (C&RT) method in order to choose the best predictor for MPO. p values < 0.05 were considered statistically significant.

Approval of the ethics board of the Medical Faculty of Adnan Menderes University was received.

## Results

**Fig. 2:**
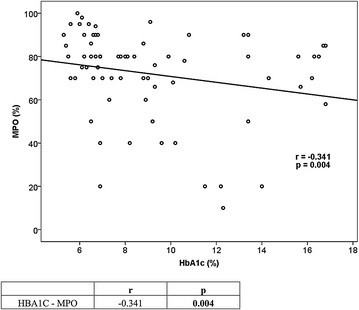
Correlation between HbA1c and MPO in patients with DM

**Fig. 3:**
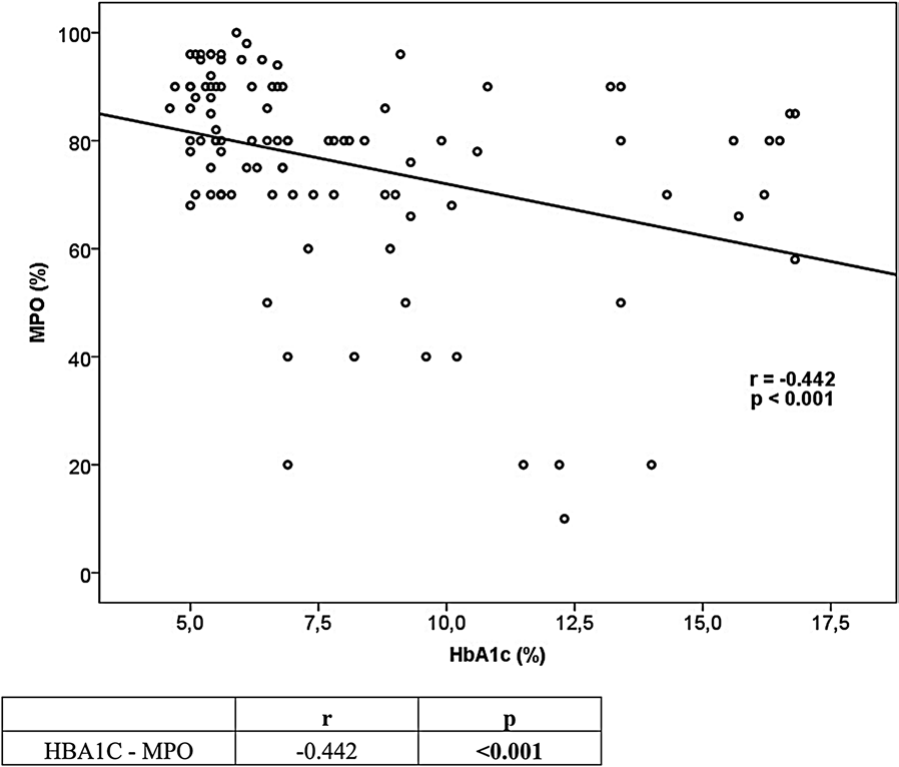
Correlation between HbA1c and MPO in all groups

Type 2 DM patients with poor glycemic control had a mean age of 56.4 ± 9.99 years and type 2 DM patients with good glycemic control had a mean age of 57.3 ± 10.02 years and healthy controls had a mean age of 51.42 ± 11.48 years, with no significant difference among the groups (p > 0.05). Type 2 DM patients with poor glycemic control had a disease duration of 12.3 ± 3.4 years and were not significantly different from type 2 DM patients with good glycemic control (p > 0.05). Myeloperoxidase ratio in neutrophils was 70 (58.5–80) in type 2 DM patients with poor glycemic control compared to 80 (73.75–90) in those with good glycemic control and 88 (78–92) in healthy controls. The DM group with poor glycemic control was statistically significantly different from the other groups (p < 0.001). Good and poor controlled diabetes groups were similar in terms of the drugs being used. No correlation was found between the MPO activity and the drugs used to treat diabetes (p > 0.05). In patient groups with diabetes, statistically significant differences in terms of MPO activity was not detected among the patients using and not using sitagliptin, insulin detemir, insulin glargine, insulin aspart, insulin lispro, insulin glulisine, metformin, gliclazide, repaglinide, nateglinide, pioglitazone. Statistical results of quantitative and qualitative data are provided in Tables 1, 2, 3 and 4; Figs. 2 and 3 demonstrates correlation analysis between MPO and HbA1c.

**Table 1:** Comparison of laboratory findings between the three groups

	DM with poor glycemic control	DM with good glycemic control	Controls	p
MPO (%)	70 (58.5–80)^a^	80 (73.75–90)	88 (78–92)	<0.001^b^
Hb (g/dL)	13.34 ± 1.63	13.52 ± 1.30	12.7 ± 1.47	>0.05^c^
Leukocyte (/mm^3^)	7961 ± 1624.48	7963.67 ± 1624.30	6608.71 ± 1411.78^d^	0.001^c^
Platelet (/mm^3^)	287375 ± 81599	280567 ± 72463	275968 ± 72470	>0.05^c^
HBA1C (%)	10.15 (8.8–13.85)	6.5 (5.98–6.8)^e^	5.4 (5–5.5)	<0.001^b^
LDL (mg/dL)	124.48 ± 42.32	113.63 ± 27.40	127.39 ± 31.67	>0.05^c^
HDL (mg/dL)	39.85 ± 11.98^f^	46.73 ± 11.91	46.29 ± 10.19	0.019^c^
Triglyceride (mg/dL)	138.5 (110.75–192.75)	137.5 (103.5–209.25)	120 (87–190)	>0.05^b^
Creatinine (mg/dL)	0.77 (0.72–0.93)^a^	0.72 (0.65–0.8)	0.73 (0.68–0.8)	0.021^b^
Fasting plasma glucose (mg/dL)	203.5 (156–304.5)	122 (97.75–128.25)^e^	90 (85–93)	<0.001^b^
Postprandial plasma glucose (mg/dL)	298 (225–397.75)	147.5 (125.5–167.75)^e^	104 (95–120)	<0.001^b^
Sedimentation (mm/h)	42.13 ± 22.27^g^	36.77 ± 18.31	26.48 ± 16.45	0.005^c^
C-reactive protein (g/dl)	3.41 (2.09–7.36)^a^	1.35 (0.7–3.85)	1.38 (0.94–3.63)	0.001^b^
Age	56.4 ± 9.99	57.3 ±10.02	51.42 ± 11.48	>0.05^c^
BMI (kg/m^2^)	29.45 (26.60–35.65)	27.88 (24.5–32.43)	25 (23.6–27.8)^d^	0.001^b^
MPV (fL)	9.4 (8.5–10.2)	8.4 (7.58–9.63)	8.9 (8.5–9.7)	>0.05^b^
Hematocrite	39.35 (37.25–42.18)	40.6 (38.58–43.03)	36.7 (35.6–41.5)^h^	0.036^b^
24-h urine microalbuminuria (mg/day)	38 (16.25–88.25)	17 (12–29.25)^e^	12 (8–15)	<0.001^b^

^a^DM group with poor control is different from other groups

^b^It was used the Kruskal–Wallis test for statistical analysis

^c^It was used the ANOVA test for statistical analysis

^d^Control group is different from other groups

^e^All groups are different from each other

^f^DM group with poor control and DM group with good control are different from each other

^g^DM group with poor control and control group are different from each other

^h^Control group and DM group with good control are different from each other

**Table 2:** Comparison of neuropathy presence and laboratory data in patients with type 2 DM

	With neuropathy (n = 27)	Without neuropathy (n = 43)	Controls (n = 31)	p
MPO (%)	75 (50–86)^a^	80 (70–90)	87 (79.5–90.5)	0.011^b^
HBA1C (%)	9 (6.9–12.3)^c^	6.75 (5.6–9.75)	5.4 (5.08–5.5)	<0.001^b^
Sedimentation (mm/h)	43.96 ± 21.89^d^	32.87 ± 20.16	31.05 ± 16.27	0.036^e^
C-reactive protein (g/dl)	4.1 (2.39–8.87)^d^	1.95 (0.83–3.88)	1.37 (0.93–4.45)	0.002^b^

^a^The group with neuropathy and the control group are different from each other

^b^It was used the Kruskal–Wallis test for statistical analysis

^c^All groups are different from each other

^d^The group with neuropathy is different from other groups

^e^It was used the ANOVA test for statistical analysis

**Table 3:** Comparison of nephropathy development and laboratory data in patients with type 2 DM

	With nephropathy (n =29)	Without nephropathy (n = 41)	Controls (n =31)	p
MPO (%)	80 (70–85)	75 (54–88)	88 (78–92)^a^	0.003^b^
HBA1C (%)	9 (7.1–12)	6.9 (6.15–10.8)	5.4 (5–5.5)^a^	<0.001^b^
Sedimentation (mm/h)	38 ± 21.25	40.39 ± 20.63	26.48 ± 16.45^c^	0.011^d^
C-reactive protein (g/dl)	3.44 (1.5–7.93)	2.41 (0.99–4.1)	1.38 (0.94–3.63)^e^	0.024^b^

^a^Control group is different from other groups

^b^It was used the Kruskal–Wallis test for statistical analysis

^c^Control group and the group without nephropathy are different from each other

^d^It was used the ANOVA test for statistical analysis

^e^Control group and the group with nephropathy are different from each other

**Table 4:** Comparison of complications presence in patients with type 2 DM

		Controls n (%)	DM with poor control n (%)	DM with good control n (%)	Exact p
Sex	Male	8 (25.8)	21 (52.5)	11 (36.7)	>0.05^a^
	Female	23 (74.2)	19 (47.5)	19 (63.3)	
Neuropathy	No		20 (50)	23 (76.7)	<0.001^a^
	Yes		20 (50)	7 (23.3)	
CAD	No		32 (80)	28 (93.3)	<0.001^a^
	Yes		8 (20)	2 (6.7)	
Retinopathy	No		31 (77.5)	28 (93.3)	<0.001^a^
	Yes		9 (22.5)	2 (6.7)	
PAH	No		38 (95)	29 (96.7)	<0.001^a^
	Yes		2 (5)	1 (3.3)	
Diabetic foot	No		38 (95)	30 (100)	>0.05^a^
	Yes		2 (5)	0 (0)	

Fisher test were used in analyzing categorical variables and descriptive statistics were expressed as frequency (%)

^**a**^It was used the Chi square test for statistical analysis

We apllied the C&RT model in order to choose the best predictor for MPO development. In the model, patients were first divided into two branches according to their type 2 DM values (patients with poor glycemic control and good glycemic control and healthy controls) for the prediction of MPO. According to this model, type 2 DM patients with poor glycemic control had lower MPO activity in neutrophils (66.6 ± 21.6 versus 81.9 ± 14.0). MPO impact on the diagnosis of Type 2 DM was 100 % and the effects of drugs used to treat DM and HT was 63 %.

## Discussion

We determined histochemically that type 2 DM patients with poor glycemic control had lower MPO activity in neutrophils compared to both healthy controls and type 2 DM patients with good glycemic control. Type 2 DM patients with good glycemic control and healthy controls did not differ significantly with respect to MPO activity in neutrophils.

Myeloperoxidase deficiency in neutrophils can be acquired or hereditary. Acquired MPO deficiency is rarer [10]. Hematologic malignancies, common cancers, cytotoxic agents, some antiinflammatory drugs, iron deficiency, lead intoxication, thrombotic conditions, renal transplantation, serious infections, neuronal lipofuscinosis, pregnancy and DM have been shown among the causes of acquired MPO deficiency [8, 10]. Nita et al. showed that fenofibrate reduced plasma MPO concentrations [16]. A study that compared MPO ratios in peripheral neutrophils in diabetic patients identified no significant difference in MPO ratios of diabetic patients with and without infection [11]. In the study by Sato et al. [12], decreased MPO activity was shown in leukocytes of diabetic patients. Likewise, a study by Uchimura et al. [17] demonstrated markedly decreased MPO activity in leukocytes of patients with non-insulin dependent diabetes. On the other hand, Gorudko et al. [13] found increased MPO activity in plasma of type 2 DM patients with no cardiovascular disease. In the study by Moldoveanu et al. [18], patients with DM also had a higher MPO activity compared to the control group. In another study, serum MPO levels were found to be higher in overweight individuals who had first-degree relatives with type 2 DM compared to the control group [19]. An animal study demonstrated that MPO expression was higher in diabetic neutrophils but reported decreased MPO activity in neutrophils compared to the control group [20].

The relationship between type 2 diabetes mellitus and MPO has been investigated by previous studies, which arrived at contradicting conclusions [13, 17–19]. There are a limited number of studies investigating the relationship between glycemic response and MPO. The study by Sato et al. evaluating the effect of glycemic response on MPO demonstrated a significant correlation between decreased MPO activity in leukocytes and increased HbA1c [12]. In our study, we also found a negative correlation between Hba1C values and MPO levels.

Previous studies investigating the relationship between DM and MPO used the ELISA method [12, 13, 17–20]. With the literature scan, our study appears as the first to evaluate MPO activity in neutrophils in diabetic patients from a histochemical perspective. Histochemical evaluation of MPO activities is cheaper and easier than serological methods.

Chronic hyperglycemia increases the release of reactive oxygen species from neutrophils [21]. Neutrophil dysfunctions were shown as one of the causes of glycemic unresponsiveness in diabetic patients. Abnormalities in granulocyte chemotaxis, phagocytosis and microbicidal activities were described for patients with poorly-controlled diabetes [22]. Impaired neutrophil bactericidal function was strongly associated with poor glycemic control and improved positively with good glycemic control [23]. The chronic hyperglycemia of poorly controlled diabetes can prime neutrophils and monocytes [24].

A positive correlation between HbA1c and white blood cell levels (WBC) in patients with type 2 DM was reported in a study [25]. Patients with type 2 DM are in a state of low-degree chronic inflammation that induces hypersecretion of inflammatory factors, which results in a constantly elevated neutrophilic granulocyte count [26]. In our study, WBC levels of DM groups with poor glycemic control and good glycemic control were statistically significantly elevated from the control group (p < 0.001).

In a study, superoxide dismutase activity in the neutrophils of type 2 diabetes patients was decreased by 41 % compared to the control group. Glutathione peroxidase (GSHPx) and glutathione reductase (GR) activities of type 2 diabetic patients were 73.04 and 81.12 %, respectively, compared to controls. No differences were noted in catalase activities. It has been suggested that these findings could account for some of the mechanisms that lead to increased sensitivity of type 2 DM patients to certain infections [27].

Increased glucose levels lead to increased protein glycation through early glycation products and advanced glycation late products (AGE) in diabetic patients. This plays an important role in occurrence of complications [28]. In addition, AGE is one of the most important causes by which hyperglycemia causes cellular and tissue damage. Some AGEs occur on critical protein sites and may lead to enzymatic inactivation and loss of physiological function [29]. Thus, decreased MPO activity in patients with poorly controlled diabetes may be due to hyperglycemia-associated negative modulation of the enzymatic activity [20].

This hypothesis was described for other enzymes previously. It suggests that the presence of high plasma glucose concentration might cause alterations in the molecular conformation of the enzyme or its catalytic site, possibly by glycation of amino acids [30].

There are varying opinions and results in the literature regarding the conditions that low MPO activity in neutrophils could lead to [31, 32]. Is MPO a friend or foe? [31]. It has been suggested that chronic inflammatory process and serious infections may be more frequent in patients with total or subtotal MPO deficiency [32]. Different publications have reported that serious infections as sporadic cases developed in less than 5 % of the individuals with MPO deficiency [8]. While it has been suggested that there might be a relationship between MPO deficiency and cancer risk [8], a different publication has reported no increase in the incidence of cancer in individuals with MPO deficiency [31]. It has been suggested that MPO deficiency has cardioprotective effects [32].

Because this was a cross-sectional study, information on patients’ infections and malignancies were derived from patient reports. The relationship between MPO deficiency and infections or malignancies could not be evaluated as conclusive data could not be obtained. We believe that evaluation by prospective studies on the clinical conditions that could be caused by MPO deficiency we observed in patients with poor glycemic control would be helpful.

There are several studies evaluating the relationship between MPO deficiency and atherosclerosis [33, 34]. In our study, a statistically significant difference was not noted in the MPO ratio between DM patients with and without neuropathy (p > 0.05). Also, no significant difference was observed between DM patients with and without nephropathy with respect to MPO levels (p > 0.05). In our study, a statistical evaluation could not be performed since the number of patients with coronary artery disease and diabetic rethinopathy in each group were insufficient. This was interpreted as a finding that differs from the findings of studies which demonstrated protective effects of deficient levels of MPO in neutrophils from atherosclerosis [33, 34]. The role of oxidative stress in development of diabetic microvascular complications is known [35] but poor glycemic control is the primary cause in development of the microvascular complications [36]. The study by Zhang demonstrated that MPO was not necessary to induce an experimental atherosclerosis model in MPO-deficiency-induced mice [37]. Our study indicates that microvascular complications in diabetic patients cannot be associated alone with MPO activity. Our study did not yield any finding which suggests that decreased MPO activity in neutrophils in diabetic patients prevented microvascular complications.

In conclusion, poor glycemic control in diabetic patients results in decreased MPO activity in neutrophils histochemically. We believe that variability in MPO activity by glycemic response in diabetic patients, complications and their clinical relevance need to be assessed in prospective studies.
